# COVID-19 Outbreak in Iran: Lessons to Learn, Measures to Take

**DOI:** 10.1017/dmp.2021.37

**Published:** 2021-02-16

**Authors:** Ata Mahmoodpoor, Sarvin Sanaie, Mohammad-Salar Hosseini

**Affiliations:** 1 Department of Anesthesiology and Critical Care Medicine, Faculty of Medicine, Tabriz University of Medical Sciences, Tabriz, Iran; 2 Neurosciences Research Center, Aging Research Institute, Tabriz University of Medical Sciences, Tabriz, Iran; 3 Student Research Committee, Tabriz University of Medical Sciences, Tabriz, Iran; 4 Research Center for Evidence-Based Medicine, Tabriz University of Medical Sciences, Tabriz, Iran

**Keywords:** COVID-19, infection control, Middle East, SARS-CoV-2

By announcing the first confirmed cases of coronavirus disease (COVID-19) on February 19, 2020, Iran joined the countries battling against the novel coronavirus. With 167 156 confirmed cases and 8134 deaths due to COVID-19 up until June 6, 2020, Iran is in the front lines of confronting this global emergency.^1^ All provinces in Iran have been affected by the virus. Considering the remarkable population density of Iran with centralization in major cities, a burst in the number of infected individuals is not far from mind. Moreover, the country was experiencing the Iranian new year (Nowruz) during the pandemic, and, due to new year celebration ceremonies, family gatherings, new year’s shopping, and holiday vacations, more exposure among the community was anticipated, which led to another peak in the number of confirmed cases.

Meanwhile, the available hospital beds and health care units are limited, and many cities may lack adequate facilities. In case of a deterioration in the situation, establishing field hospitals could be beneficial. The limitation in health care workers and the importance of their being healthy require a proper procurement and distribution of personal protective equipment (PPE). Where possible, assigning separate triage, radiology, and laboratory units to equipped centers is a practical step toward protecting non-infected patients. To improve the current situation, people should be informed to avoid unnecessary referrals to the hospitals, and elective surgeries should be recommended to be postponed. Physicians could benefit from telemedicine, helping them to manage non-critical patients remotely instead of arranging an in-person visit.^2^

Future peaks of the disease are not unexpected. In fact, the current increase in the number of confirmed cases may still count for the first peak. As the potential capacity of the health care system is approaching its limits, balancing the burden of disease and efficient management of the patients become more important. To attain this purpose, local health care units have developed approaches to handle the increasing load of patients efficiently. For instance, since prone position, as a rescue treatment, cannot be performed in our centers, we can use early application of prone position in critically ill awake, non-intubated, and hypoxemic patients to improve oxygenation and decrease the rate of intubation.^3^ Regarding the post-infection immunity of the disease, it seems that using approaches like deploying convalescent health care workers to care for confirmed COVID-19 patients could also be useful.

Following the development of social media, the risk of misinformation – especially among the less-informed populace – is serious.^4^ For this, a reliable source should be presented to follow the latest news, information, and international and national guidelines. Thereby, we should take the most advantage of cyberspace and online education tools. A valuable measure taken by the Ministry of Health and Medical Education was to publish and release national guidelines and instructions for COVID-19 diagnosis and treatment. Since new findings are presented every day, releasing continuous updates on these guidelines is an undeniable necessity.^5^ Additionally, funding for further research on early diagnosis of the disease and effective management is a wise and reasonable decision.

Considering the forthcomings, cancellation of gatherings, ceremonies, sporting events, and applying commuting restrictions inside the cities and between the cities are strongly recommended. The “social distancing” plan is officially administered throughout the country and is expected to be in effect until the number of affected people is decreased. During this plan, a complete lockdown is commanded to avoid any unnecessary transportation, which is waived step-by-step to prevent mass exposure. Another advantageous measure is the national mobilization for screening patients, using the online system (salamat.gov.ir) to screen the whole population remotely. Registered by over 69 000 000 individuals, this project reduces unnecessary referrals to health care facilities.^6^ Also, it could lead to identifying the regions with more prevalence of the disease by elucidating the geographical pattern and revealing how the disease is spreading. As most of the new cases are a cluster of people with the same limited exposures, contact tracing may help slow down the spreading of disease by identifying the clusters and prevention of disease spreading.^7^ Although airports, train stations, and bus terminal screening stations could help prevent the disease spread to other cities, setting up these stations in random places across the cities may only contribute to more gatherings and increase the risk of the virus spreading. The fact is that Iran is more focused on social distancing rather than quarantining.^8^

Since Iran has a relatively young population, the Ministry of Health and Medical Education should prioritize primary health care programs (eg, maternal and child health, antenatal, and vaccination programs) during the crisis to cope with the ongoing waves of transmissions and ready for interventions that improve patients’ outcomes.

This challenge could be a guide to prepare for future outbreaks, demonstrating the importance of implementing emergency guidelines, establishing biosafety level 4 laboratories, and providing access to sufficient PPEs. For Iran, COVID-19 is not the last epidemic to confront; however, by preparing to encounter medical states of emergency and fostering infrastructures, it could be the last one causing trouble.
